# The Effect of Diet on Improved Endurance in Male C57BL/6 Mice

**DOI:** 10.3390/nu10081101

**Published:** 2018-08-16

**Authors:** Jin Yu, Hong Zhu, Saeid Taheri, Stephen Perry, Mark S. Kindy

**Affiliations:** 1 Department of Pharmaceutical Sciences, College of Pharmacy, University of South Florida, Tampa, FL 33612, USA; jinyu@health.usf.edu (J.Y.); hongzhu@health.usf.edu (H.Z.); taheris@health.usf.edu (S.T.); 2 NutriFusion^®^, LLC, Naples, FL 34109, USA; Sperry@consealint.com; 3 James A. Haley VA Medical Center, Tampa, FL 33612, USA; 4 Shriners Hospital for Children, Tampa, FL 33612, USA

**Keywords:** diet, endurance, mitochondrial biogenesis, autophagy

## Abstract

The consumption of fruits and vegetables appears to help with maintaining an adequate level of exercise and improves endurance. However, the mechanisms that are involved in this process are not well understood. In the current study, the impact of diets enriched in fruits and vegetables (GrandFusion^®^) on exercise endurance was examined in a mouse model. GrandFusion (GF) diets increased mitochondrial DNA and enzyme activity, while they also stimulated mitochondrial mRNA synthesis in vivo. GF diets increased both the mRNA expression of factors involved in mitochondrial biogenesis, peroxisome proliferator-activated receptor gamma coactivator 1 alpha (PGC-1α), mitochondrial transcription factor A (Tfam), estrogen-related receptor alpha (ERRα), nuclear respiratory factor 1 (NRF-1), cytochrome c oxidase IV (COXIV) and ATP synthase (ATPsyn). Mice treated with GF diets showed an increase in running endurance, rotarod perseverance and grip strength when compared to controls who were on a regular diet. In addition, GF diets increased the protein expression of phosphorylated AMP-activated protein kinase (AMPK), sirtuin 1 (SIRT1), PGC-1α and peroxisome proliferator-activated receptor delta (PPAR-δ), which was greater than exercise-related changes. Finally, GF reduced the expression of phosphorylated ribosomal protein S6 kinase 1 (p-S6K1) and decreased autophagy. These results demonstrate that GF diets enhance exercise endurance, which is mediated via mitochondrial biogenesis and function.

## 1. Introduction

The maintenance of regular physical activity throughout life is important for a healthy existence and long-term viability in humans [1]. Exercise is dependent upon the generation of ATP, which is produced by mitochondria in the cells [2]. An increase in the mitochondrial number and performance is critical in improving physical performance and overall well-being [3]. Studies have shown that exercise increases mitochondrial biogenesis and oxidative capacity, which specifically occurs in skeletal muscle [4].

The skeletal muscle is responsible for the skeletal movement and is composed of slow (type I) and fast (type II) muscle fibers [5]. Type I and type IIa fibers undergo oxidative metabolism, are rich in mitochondria and are fatigue resistant [6]. Slow-twitch muscle fibers generate less power and strength than fast-twitch fibers, but they can sustain activity for longer [7]. Fast-twitch muscle fibers generate far more power and strength, but they fatigue much faster and require more time for recovery. The slow-twitch fibers are metabolically adaptive and can improve mitochondrial function and outcomes under specific conditions [8].

GrandFusion^®^ (GF) diets contain a mixture of fruits and vegetables, which are highly enriched in vitamins that are able to limit the extent of cerebral ischemia injury and reverse several parameters of stroke, such as inflammation, oxidative stress and behavioral changes [9]. Additionally, GF extracts have been shown to improve memory and learning in aged rats, which is mediated by antioxidant enzymes and signaling pathways [10]. Previous studies have shown that GF diet has anti-inflammatory, anti-oxidant, neuroprotective and neurogenic properties [9,10].

In the present study, we aimed to determine the impact of diets that are rich in vegetables and fruits on the physiological and biochemical benefits on stamina and endurance. Mice were fed diets that were enriched in fruits and vegetables for 20 weeks, before being examined. The results revealed that these diets enhanced physical performance in a number of different behavioral assays by altering muscle parameters. We have demonstrated that the supplemented diets enhanced mitochondrial biogenesis and enhanced endurance through the activation of AMP-activated protein kinase (AMPK), sirtuin 1 (SIRT1), peroxisome proliferator-activated receptor gamma coactivator 1 alpha (PGC-1α) and peroxisome proliferator-activated receptor delta (PPAR-δ). These data suggest that these diets can influence the physical performance.

## 2. Materials and Methods

### 2.1. Animal Experiments

Ten-week-old male C57BL/6J mice (Jackson Laboratories, Bar Harbor, ME, USA) were housed in a controlled environment (25 °C ± 2 °C, 55 ± 5% relative humidity with a 12-h light/12-h dark cycle) at the Medical University of South Caolina animal facility. The mice were randomly divided into three groups: Group 1 received a normal chow diet (Harlan Teklad Global 18% Protein Rodent Diet (#2018), Con); Group 2 received a 2% GrandFusion (GF1, NF-216—Fruit and Veggie #1 Blend) with the normal chow; and Group 3 received a 2% GrandFusion diet (GF2, NF-316—Fruit #2 Blend) with normal chow provided by NutriFusion, LLC (Naples, FL, USA) (www.nutrifusion.com), which was described previously [9,10]. Table S1 shows the diets used in the studies (supplemental material). At the end of the 20-week oral administration period, the exercise endurance of all mice was measured before the mice were euthanized. This study adhered to the Guide for the Care and Use of Laboratory Animals and was approved by the VA IACUC (#351). All animals were randomized to the groups and were assessed by researchers, who were blinded to the allocation of animals. No animals were excluded from the studies due to adverse events.

### 2.2. Endurance Testing

Grip power reflecting the total power of four limbs of the mice was measured using a standard grip strength meter for mice (San Diego Instruments, San Diego, CA, USA). The mice were put on a metal mesh and pulled horizontally. The grip power was defined as the power of traction when the mice released the mesh. Measurements were repeated three times and the data were averaged. Running distance was measured on a mouse treadmill (Omnitech Electronics, Inc., Columbus, OH, USA) according to a previously described protocol [11,12]. Mice were forced to run on the motor-driven treadmill until they were completely exhausted, which was defined as the point at which they remained on the electrical shocker plate for more than 30 s. The treadmill was set at an incline of 10%. The speed was set to be 18 cm/s at the beginning and was increased by 3 cm/s every 2 min, which followed three days of acclimation running at 18 cm/s for 5 min. The average running time until exhaustion for wild-type mice on normal chow was ~55 min. For measuring fine motor coordination and stamina, rats were placed on an accelerating rotarod (San Diego Instruments, San Diego, CA, USA), which consisted of a slowly accelerating (+2 rpm/30 s; 20 rpm max) rotating dowel (7 cm diameter). Their latency to fall was recorded (max 400 s).

### 2.3. Reverse Transcription-Polymerase Chain Reaction

Total RNA was isolated from the soleus muscle using TRIzol reagent (ThermoFisher-Invitrogen, Carlsbad, CA, USA), before being converted to cDNA with Reverse Transcription Premix (ThermoFisher-Applied Biosystems, Waltham, MA, USA). To quantify mRNA expression, the cDNA was amplified with SuperScript VILO PCR PreMix (ThermoFisher-Applied Biosystems, Waltham, MA, USA) and primer pairs (IDT, Coralville, IA, USA). PCR was performed using a iCycler PCR System (Bio-Rad, Hercules, CA, USA). PCR products were resolved using 1.5% agarose gel electrophoresis and visualized on a DigiDoc-it Imaging System (UVP, Upland, CA, USA). β-Actin was used as an internal control. The primers used are listed in Supplemental Materials.

### 2.4. Western Blot Analysis

Skeletal muscle tissues were homogenized and lysed using radioimmunoprecipitation assay RIPA lysis buffer (Boston Bioscience, Boston, MA, USA) with a protease inhibitor cocktail (Sigma-Aldrich, St. Louis, MO, USA). The lysate protein concentrations were determined by the Bradford ELISA assay. The primary antibodies used were phosphor-AMPK, AMPK, SIRT1, β-actin (1:1000; Cell Signaling, Beverly, MA, USA), PGC-1α, PPAR-δ, phosphor-S6K1 and S6K1 (1:1000; Santa Cruz Biotechnology, Inc., Santa Cruz, CA, USA). Bound antibodies were detected using horseradish peroxidase-linked secondary antibodies (1:5000; Molecular Probes/Invitrogen, Eugene, OR, USA) for 2 h. Proteins were detected with the enhanced chemiluminescence (ECL) detection system (GE Life Sciences/Amersham Biosciences, Pittsburgh, PA, USA) and visualized using the DigiDoc-it Imaging System (UVP, Upland, CA, USA). β-actin was used as an internal control.

### 2.5. Examination of Mitochondrial Amount and Enzyme Activity in Muscle

Tissue samples from gastrocnemius and soleus muscles were obtained at the age of 20 weeks for physiological and molecular biological analysis. The mitochondria were isolated from tissue by a density gradient centrifugation method (ThermoFisher, Waltham, MA, USA). Tissue samples were homogenized in a mitochondria isolation buffer and mitochondria were isolated as previously described [3]. The activity of cytochrome *C* oxidase (COX) was quantified by mixing isolated mitochondria and ferrocytochrome *C*, while the absorption at 550 nm was measured to determine the COX activity of the samples (Invitrogen, Camarillo, CA, USA). The activity of β-hydroxyacyl-CoA dehydrogenase (β-HAD) was determined by a previously established method [3]. The activity of citrate synthase (CS) was measured (Sigma-Aldrich, St. Louis, MO, USA). 

### 2.6. Analysis of Mitochondrial DNA Content

Total RNA was isolated from skeletal muscle tissue using TRIzol reagent (Invitrogen, Eugene, OR, USA), and converted to cDNA. The results were expressed as the relative number of mitochondrial genomes per diploid nuclei. The primers used are listed in Supplemental Materials. The ratio of mtDNA and genomic DNA was determined by measuring the relative density of the band.

### 2.7. Adiponectin Enzyme-Linked Immuno Sorbent Assay (ELISA)

Plasma adiponectin levels were determined by ELISA kits (MRP300, R&D Systems, Minneapolis, MN, USA).

### 2.8. Statistical Analysis

The results were expressed as the mean ± standard deviation (SD). The statistical significance of the results in the RNA, behavioral studies, physiological and biochemical data was analyzed using a *t*-test or one-way analysis of variance (ANOVA) followed by Tukey’s post hoc test. Repeated-measures ANOVA were calculated for the monitoring data and the significance of the differences between groups were evaluated by Tukey’s post hoc test.

## 3. Results

### 3.1. Diets, Food Intake and Weight

**Figure 1:**
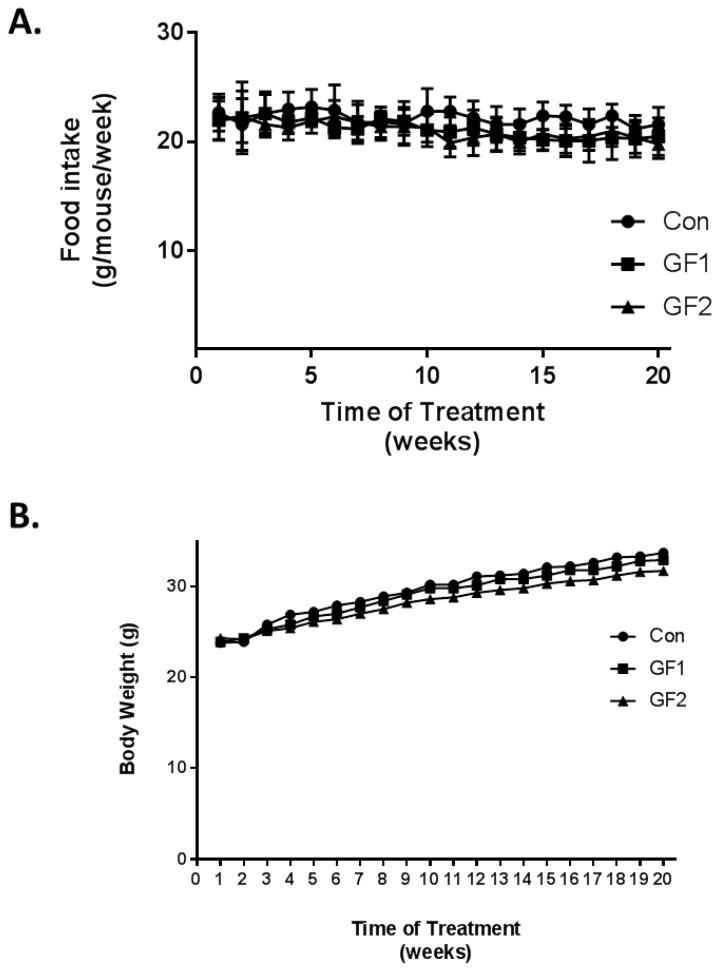
Effects of GF diets on food intake and body weight. (**A**) Food intake changes in 10 weeks. (**B**) Body weight changes in mice on various diets over 10 weeks. Mice were fed a normal diet or diets that were supplemented with 2% GF. Each point represents mean ± SD (*n* = 10 per group).

Ten-week-old mice were fed diets, which were supplemented with GrandFusion diets (2%) for 20 weeks. The diets were as follows: Group 1 received the normal diet alone; Group 2 received a 2% GrandFusion (GF1, NF-216—Fruit and Veggie #1 Blend) with the normal diet; and Group 3 received a 2% GrandFusion diet (GF2, NF-316—Fruit #2 Blend) with the normal diet. These are same diets that were used in previous studies [9,10]. The animals were examined for food intake and body weight every week for the twenty weeks of feeding. As seen in Figure 1A, the mice on all diets maintained a constant intake of food over the course of the study. In addition, consistent with the food intake, all of the mice showed a similar gain in weight over the twenty weeks, although the GF1 and GF2 mice only showed a modest increase in body weight.

### 3.2. Exercise and Endurance

**Figure 2:**
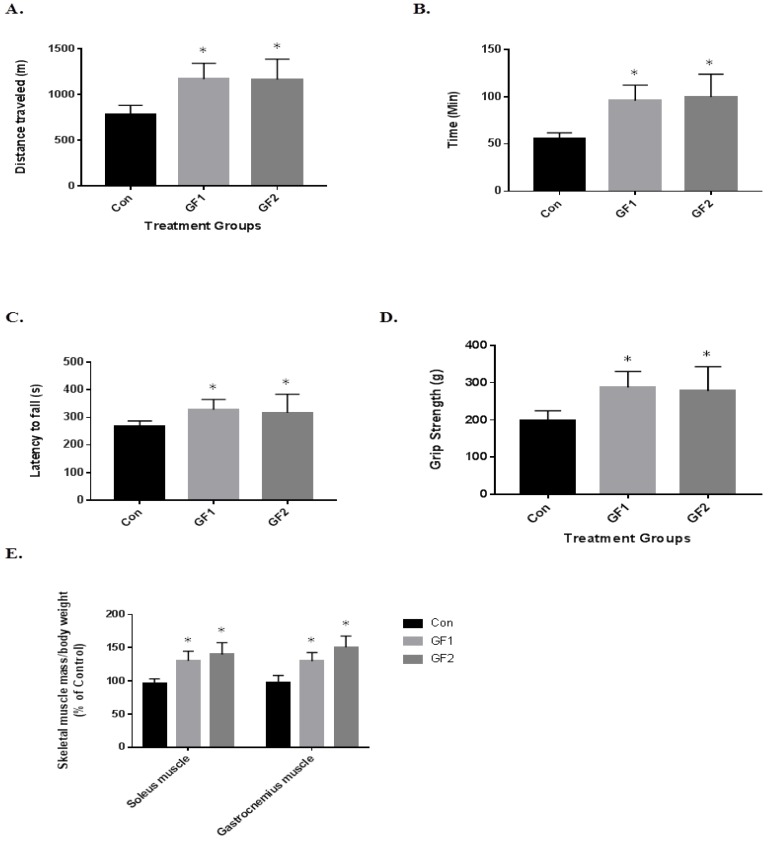
Effects of GF diets on exercise endurance and skeletal muscle mass in C57BL/6 mice. (**A**) Running distance and (**B**) time of normal mice and mice that were fed a normal diet enriched with GF supplements. (**C**) Time to fall in a rotarod test (**D**) grip strength and (**E**) the ratio of skeletal muscle mass (soleus and gastrocnemius muscle)/body weight in normal and GF supplemented mice. Data are expressed as the mean ± SD (*n* = 10 per group), * *p* < 0.01 compared to control group.

To determine the impact of the diets on exercise endurance and activity, the mice were subjected to several paradigms (Figure 2). In Figure 2A, the mice on both the GF1 and GF2 supplemented diets showed a 1.5-fold increase in the distance traveled compared to the control diet mice (Figure 2A). In addition, the GF1 and GF2 mice demonstrated a 1.7-fold and 1.8-fold increase in time on the treadmill (Figure 2B). When the mice were subjected to the rotarod testing, the mice showed a 1.2-fold increase in the time to fall for both the GF1 and GF2 supplemented diets when compared to the control diet (Figure 2C). When the GF1 and GF2 were compared to the control diet for grip strength, there was a 1.45-(GF1) and a 1.40-fold (GF2) increase. Finally, both the soleus (SM) and gastrocnemius (GM) muscles were removed from the mice after the twenty-week treatment. The GF treated mice showed a 1.33-(SM) and a 1.45-fold (GM) increase for the GF1 diet, while there was a 1.32-(SM) and 1.55-fold (GM) increase after the GF2 diet. These results show that the GF supplemented diets significantly increased exercise endurance in the mice compared to the control diet animals. As indicated above, the mice showed a modest increase in body weight, which is consistent with the increase in muscle mass. In addition, a decrease in fat might account for the changes (not determined).

### 3.3. Impact on Mitochondrial Function

**Figure 3:**
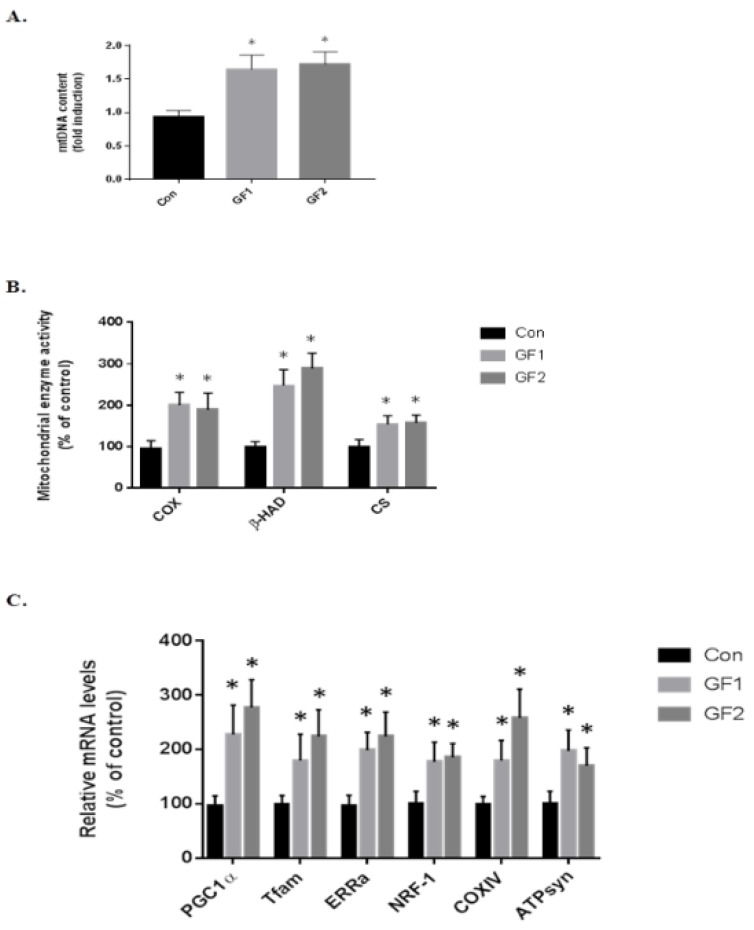
Effect of GF diets on muscle mitochondrial biogenesis in C57BL/6 mice. (**A**) The amount of mitochondrial DNA (mtDNA) in mice that were fed a control diet or a diet enriched in GF as determined by the mtDNA/genomic DNA ratio. (**B**) The activity of mitochondrial enzymes COX, β-HAD and CS were evaluated as a percent of control. (**C**) The relative mRNA levels of PGC-1α, Tfam, ERRα, NRF-1, COXIV and ATPsyn in controls and GF fed mice. The results are expressed as the mean ± SD (*n* = 10 per group), * *p* < 0.01 compared to the control group.

To determine the role of the skeletal muscle cell mitochondria in increasing endurance in the mice treated with GF diets, at the end of the study, the skeletal muscle was removed and examined for mitochondria DNA content at the end of the study (mtDNA, Figure 3A). As seen in the figure, there was an increase of 1.76-(GF1) and 1.85-fold (GF2) compared to the control diet fed mice. Figure 3B shows the changes in mitochondrial enzymes. The protein was isolated and subjected to enzymatic analysis. When mice were treated with the GF diets, there were significant increases in cytochrome c oxidase (COX), β-hydroxyacyl-CoA dehydrogenase (β-HAD) and citrate synthase (CS) (Figure 3B). As seen in the figure, COX increased 2.0-(GF1) and 1.9-fold (GF2); β-HAD was increased 2.5-(GF1) and 2.9-fold (GF2); and CS was increased 1.5-(GF1) and 1.6-fold (GF2) compared to control mice. Finally, we analyzed several mitochondrial mRNAs to establish the role of GF supplementation on mitochondrial function (Figure 3C). The increases in mRNA expression were: PGC-1α–2.3-(GF1) and 2.8-(GF2); Tfam–1.8-(GF1) and 2.3-(GF2); ERRα–2.0-(GF1) and 2.3-(GF2); NRF-1–1.8-(GF1) and 1.85- (GF2); COXIV–1.8-(GF1) and 2.6-(GF2); ATPsyn–2.0-(GF1) and 1.7-(GF2) fold. These results demonstrate an increase in mitochondrial activity and function when the mice were given GF diets. There were no significant differences between the GF groups.

### 3.4. Diet Impact on Exercise Signaling in Skeletal Muscle

**Figure 4:**
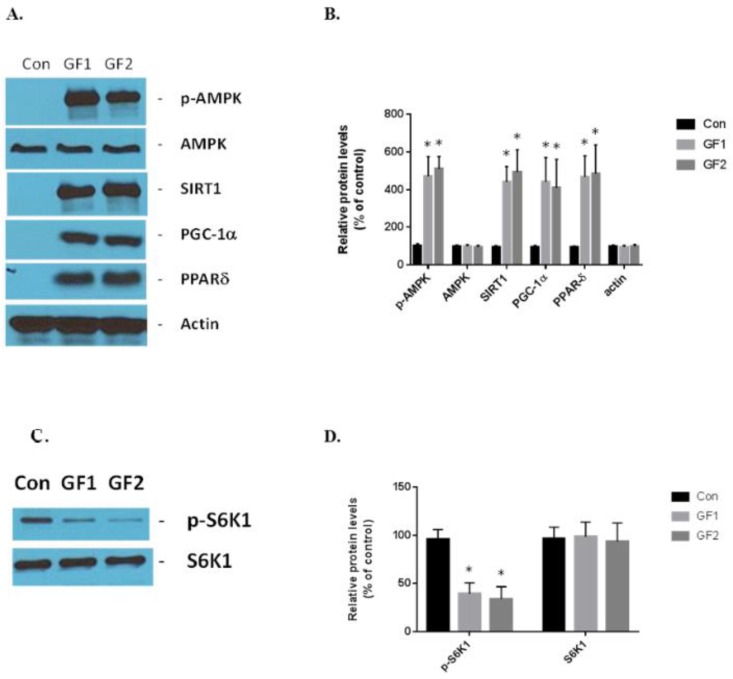
The effect of GF diets on the exercise signaling pathways and autophagy. (**A**) The protein expression of exercise-associated markers, such as p-AMPK, AMPK, SIRT1, PGC-1α and PPARδ, was evaluated by Western blot analysis. (**B**) The protein expression levels in (**A**) were plotted for statistical analysis. (**C**) Mice that were fed control or GF diets were evaluated for p-S6K1 and S6K1 activity. (**D**) Evaluation of data from (**C**). The results are expressed as mean ± SD (*n* = 10 per group), * *p* < 0.01 compared to the control group.

The GF supplemented diets stimulate exercise signaling in the skeletal muscle cells. AMPK, SIRT1, PGC-1α and PPARδ are the critical sensors and regulators of energy expenditure and biogenesis in the mitochondria [4]. GF diets increased the expression of phosphorylated AMPK, SIRT1, PGC-1α and PPARδ in vivo compared to the control mice (Figure 4A,B). The increases in protein expression were: pAMPK–4.7-(GF1) and 5.1-(GF2); SIRT1–4.4-(GF1) and 5.0-(GF2); PGC-1α–4.4-(GF1) and 4.1-(GF2); and PPARδ–4.7-(GF1) and 4.85-(GF2) fold. Finally, we examined the expression of S6K1 and its phosphorylated form in the presence of GF supplemented diets (Figure 4C,D). We found that p-S6K1 decreased by 60% and 67% after treatment with GF1 and GF2 diets, respectively, compared to the control diets. These data support the hypothesis that the GF supplemented diets enhance mitochondrial biogenesis and function while suppressing autophagy [13].

### 3.5. Diet Induced Adiponectin Levels in Plasma

**Figure 5:**
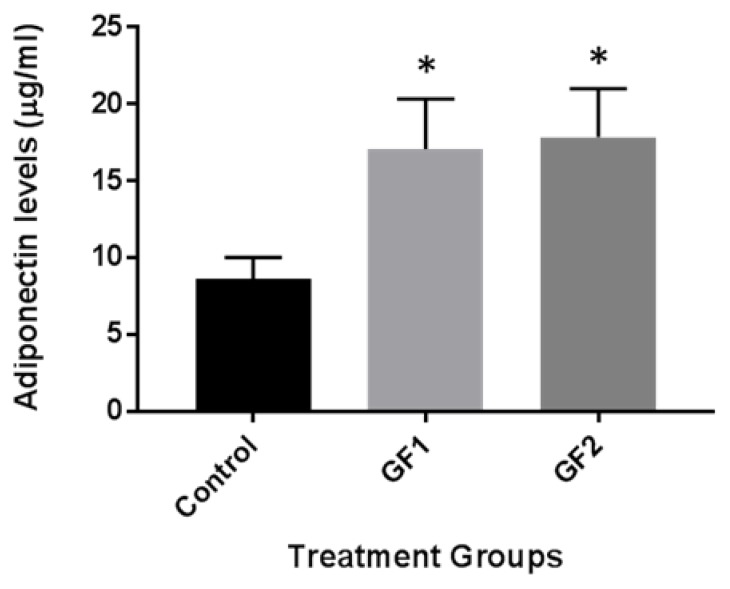
The effect of GF diets on the exercise signaling pathways and autophagy. The expression of GF/exercise-associated adiponectin levels were evaluated by ELISA. Mice that were fed control or GF diets were evaluated for adiponectin levels. The results are expressed as mean ± SD (*n* = 10 per group), * *p* < 0.01 compared to the control group.

Finally, we examined the impact of the different diets (GF1 and GF2) on adiponectin levels in the plasma of the mice. Recent studies with caloric restriction showed an increase in adiponectin levels and promoted blood flow recovery following hindlimb ischemia in mice subjected to exercise [14,15,16]. The mice, which were fed control chow or chow with the diets, were examined for adiponectin levels. As seen in Figure 5, adiponectin levels were increased in the mice that were fed with GF1 and GF2 diet compared to the mice that were given control chow. These data suggest that GF regulation of adiponectin may have cardiovascular benefits that help to enhance the outcomes of enhanced function.

## 4. Discussion

In the present study, we examined how diets that are rich in vegetables and fruits can have an effect on the control of mitochondrial function in skeletal muscle and found that long-term feeding of these diets for 20 weeks increased skeletal muscle mitochondria and mitochondrial function, which enhanced the exercise endurance.

Previous studies have shown that a number of factors are important in the regulation of exercise endurance, which is mediated through mitochondrial function [17,18]. Research has implicated PGC-1α in controlling mitochondrial biogenesis and linking several processes that are associated with energy metabolism [19]. As part of this relationship, PGC-1α is known to cooperate with NRF-1 to activate the expression of Tfam. Increased expression of Tfam has been shown to mediate the replication of mitochondrial DNA, activate transcription and orchestrate oxidative phosphorylation in skeletal muscle [20]. PGC-1α stimulates the expression of ERRα, which is important for the regulation of fatty acid oxidation and enzymes in the oxidative phosphorylation pathway [21]. The GF supplemented diets can stimulate the expression of PGC-1α, ERRα, NRF-1 and Tfam mRNA [22]. These data further support the hypothesis that nutritional supplements can increase the total mitochondria by the stimulation of mitochondrial biogenesis [23]. We speculate that nutritional enhancements can adjust endurance during exercise by intensifying mitochondrial behavior. Studies have demonstrated that mitochondrial production and performance are reduced in the aging population due to genetic defects in mitochondrial function. Furthermore, alterations in mitochondrial activity are associated with age-related disorders [24,25]. The impact of nutritional supplements on the specific roles of mitochondrial may transcend many different physiological states [26].

As indicated above, AMPK, SIRT1, PGC-1α, ERRα, NRF-1 and PPARδ all have important responsibilities in energy metabolism [20]. In addition, they are all enhanced by exercise and various diets [12,20]. Recent studies have shown that the overexpression and deletion of these factors contributes to the regulation of exercise endurance and that stimulation of these entities is critical to improve physical endurance [27]. Transgenic PGC-1α mice have been shown to regulate and coordinate factors that are involved in skeletal muscle function and hypertrophy [28]. PGC-1α increases the expression of genes associated with red fibers, mitochondrial function, fatty acid oxidation and branched chain amino acid (BCAA) degradation [29,30]. In addition, studies have shown that the purine nucleotide pathway, malate–aspartate shuttle and creatine metabolism are elevated by PGC-1α, further characterizing its role in exercise metabolism. The overexpression of the metabolic sensor and regulator SIRT1 in skeletal muscle demonstrated a fiber shift from fast-to-slow twitch, increased levels of PGC-1α, oxidative metabolism and mitochondrial biogenesis [31]. We demonstrated that GF supplementation activated transcription of both mRNA and protein of AMPK, SIRT1, PGC-1α, ERRα, NRF-1 and PPARδ in the signal cascade of exercise. In addition, the increase in adiponectin in the plasma of GF treated animals suggests an alteration in the blood flow that might help enhance the outcomes seen in the study [14,15,16]. 

Our previous studies have demonstrated the impact of nutritional supplements in improving recovery from neurological disorders as well as attenuating age-related declines [9,10]. We showed that supplementation of GF diets prior to cerebral ischemic injury in mice attenuated the damage in the brain following the injury [9]. In addition, the diets reduced the levels of oxidative stress, inflammation and induced neurogenesis. In the aged rat model, the diets reduced oxidative stress and inflammation and improved physical performance in the aged animals [10]. The presence of vegetables and fruits containing phytonutrients are essential for prevention or reducing the risk of disease as well as tempering the outcomes following injury or disease. 

## 5. Conclusions

In summary, this study demonstrated that the long-term treatment of animals with diets enriched with vegetable or fruit extracts increased muscle mitochondria via the activation of AMPK pathways. These data suggest that the increase in mitochondria in the muscle is instigated by the diets. These diets contain antioxidants, anti-inflammatory agents, nutrients and factors, which provide the increase in exercise endurance. Therefore, since the muscle mitochondria are dynamic elements that help to enhance physical implementation, approaches that stimulate and invigorate mitochondrial viability are necessitated to continue the process of exercise endurance.

## Supplementary Materials

The following are available online at http://www.mdpi.com/2072-6643/10/8/1101/s1, Table S1: Composition of GrandFusion Supplements.

## Abbreviations:

AMPK	AMP-activated protein kinase
ERRα	estrogen related receptor α
GF	GrandFusion
NRF-1	nuclear regulatory factor 1
PGC-1α	peroxisome proliferator-activated receptor gamma coactivator 1 α
PPARδ	peroxisome proliferator-activated receptor δ
SIRT1	sirtuin 1
Tfam	mitochondrial transcription factor A
